# Adolescents and dual pathology. Assessment of treatment with paliperidona palmitato long-term injectables

**DOI:** 10.1192/j.eurpsy.2021.618

**Published:** 2021-08-13

**Authors:** S. Pérez Sánchez, I. Martín Herrero, M.A. Cutillas Fernández, D. Raya Güimil, A. Crespo Portero

**Affiliations:** 1 Servicio De Psiquiatría, Servicio Murciano de Salud, Murcia, Spain; 2 Servicio De Psiquiatría, Servicio Murciano de Salud, Lorca, Murcia, Spain

**Keywords:** Dual pathology, adolescent, cocaine, borderline personality disorder

## Abstract

**Introduction:**

Today cocaine use is very frequently associated with adolescents with maladaptive personality traits and impulse control disorder. It requires a multidisciplinary approach and individualized treatments to improve the clinic and achieve the abandonment of consumption.

**Objectives:**

1. To assess the efficacy of monthly injectable paliperidone palmitate treatment in controlling impulsivity. 2. Determine the consumption of toxins after treatment.

**Methods:**

Sample: Adolescents, 14-17 years old, with a diagnosis of Personality Limit T and cocaine consumption who start treatment with Paliperidone Palmitate LD IM (50-100mg / month) in monotherapy, with Diazepam 5mg if significant anxiety. Retrospective data collection. Plutchik impulsivity scale (IE) before starting treatment and at 3 months. Statistical analysis SPSS 20.0

**Results:**

Twelve adolescents who met the inclusion criteria were included and 12 adolescents, 83% male, 16% female, completed the questionnaires. After its application and correction through non-parametric tests (N <30), scores in the EI questionnaire of a mean of 37.42 points in the pretest were observed, corresponding to a severe level of impulsivity; and a mean of 26.28 points in the post-test, compatible with a mild-moderate degree of impulsive symptoms. A decrease in the consumption of toxins was observed in 65% of the cases.

**Conclusions:**

In our experience, the management of toxic consumption in adolescent population with severe impulsivity symptoms has great limitations due to the scarce resources available. The Palpitate of Paliperidone long-term treatment has been useful in the approach of serious registered cases, being associated with symptomatic improvement and decrease in consumption.

